# Exploring knowledge of parents and caregivers on cancer symptoms in children: an observational study regarding the need for educational tools and health promotion in low- and middle-income countries

**DOI:** 10.1186/s12887-022-03686-4

**Published:** 2022-11-04

**Authors:** Leslie V. Uribe-Ortiz, Bárbara M. Garza-Ornelas, Ana C. Vázquez-Fernández, Fabiola Castorena-Torres, Julieta Rodríguez-De-Ita

**Affiliations:** Tecnologico de Monterrey, Escuela de Medicina y Ciencias de la Salud, Ave. Morones Prieto 3000, 64710 Monterrey, Nuevo Leon Mexico

**Keywords:** Cancer symptoms, Caregivers, Childhood cancer, Children, Education, Health promotion, Knowledge, Parents

## Abstract

**Background:**

Although most cases of childhood cancer are unlikely to be prevented, by today’s standards, most children with cancer can now be cured. However, disparities about survival exist among countries; in Mexico, the overall survival is 49.6%, with 70% of childhood cancers diagnosed at advanced stages. Therefore, parents and caregivers must have optimal knowledge of the early signs and symptoms of childhood malignancies as they are largely non-specific. This study was designed to explore the current knowledge of childhood cancer among parents and caregivers in Mexico and identify the need for education and health promotion in low- and middle-income countries.

**Methods:**

An online survey of 112 parents and caregivers was performed to assess their knowledge of childhood cancer, focusing on the signs and symptoms and early diagnostic strategies.

**Results:**

Sixty-nine (61.6%) mothers, 23 (20.5%) fathers, 17 (15.2%) familiar caregivers, and 3 (2.7%) non-familiar caregivers responded. Forty-six (41.1%) respondents said that they knew a child diagnosed with cancer, 92.9% mentioned leukemia as the most common type of cancer among children, the most highly ranked option when asked which sign/symptom they considered as a warning for suspicion was growth/lump in any part of the body, 97.3% considered that an early diagnosis is related to a higher cure rate, and 92.9% expressed the desire to receive reliable information about childhood cancer.

**Conclusions:**

Although parents and caregivers have some knowledge of childhood cancer, there are concepts that should be reinforced to improve their understanding of this group of diseases, as they are the frontline for children to seek medical attention. In the future, the use of tools that help educate more caregivers will strengthen knowledge and contribution regarding this issue and promote the generation of public policies that support the early diagnosis of childhood cancer.

## Background

Cancer is a global health problem in both the pediatric and adult populations. Recently, the diagnosis of this group of diseases has increased in the pediatric population because of several factors. Nevertheless, making an early diagnosis is of great importance, which is crucial for treatment and the improvement of patient outcomes, a critical factor in low- and middle-income countries (LMICs).

There are specific challenges unique to LMICs that result in pediatric cancer diagnostic delays. However, only a few articles have been published in developing countries about early diagnosis [1–4]. These specific challenges include cultural and socioeconomic differences. Moreover, according to other authors, other factors related to diagnostic delay were patient-related (e.g. age, sex, family size, parental education, residence, socioeconomic level, type of cancer, and presenting symptoms), physician-related, or health system-related. A study conducted in 2022 in Mexico reported that children whose parents had the lowest educational level had longer diagnostic delays than those children with parents with the highest educational level [4]. Of these factors, parental knowledge and education are important modifiable factors that we can address, and studies have reported that parental knowledge and education may or may not be related to cancer diagnostic delays in children [5–8].

Although most cases of childhood cancer are unlikely to be prevented, by today’s standards, most children with cancer can now be cured [9, 10]. Unfortunately, substantial disparities in survival rates exist among countries, as 94% of all cancer deaths worldwide among children occur in LMICs [10].

In Mexico, the overall survival rate for childhood cancer is 49.6%, with variability ranging from 6.8% to 64.1% among states [11]. Moreover, approximately 75% of cases are diagnosed at advanced stages [12], an important risk factor that has a profound impact on patient survival [9].

Because the signs/symptoms of cancer in children are largely non-specific, it is essential that parents and caregivers have optimal knowledge regarding the early signs/symptoms of childhood malignancies [8, 9]. Therefore, this study was designed to characterize the current knowledge of childhood cancer among a population of parents and caregivers in Mexico.

## Methods

### Setting and study population

We conducted a one-time survey involving Mexican parents and caregivers of children and adolescents of the ages 0–18 years, who attended an education and child development forum named “ExpoLearning Kids in January 2020. The forum was attended approximately by 600 individuals, of whom 112 parents and caregivers, who were personally invited, agreed to participate in this study. None of the 112 parents and caregivers declined to participate. This forum focused on providing information based on lectures and debates with parents and caregivers about child and youth education, health, nutrition, mindfulness, and physical and artistic activities. This forum was held in Monterrey, Nuevo Leon, Mexico, which includes its metropolitan area (composed of 13 cities), and is the second largest metropolitan area in Mexico, with an approximate population of 5 million inhabitants [13]. The Mexican health system comprises two sectors: public and private. In Monterrey, public healthcare is delivered by several institutions: [14, 15] (1) the Mexican Social Security Institute provides compulsory health insurance to workers in the formal labor market and their families (68 million individuals in Mexico and 4.4 million individuals in Nuevo Leon); (2) the Secretary of Health, the National Institutes of Health, and the secretaries of health at the state level provide care to individuals affiliated to the Social Protection System in Health through its operating arm Seguro Popular (54 million individuals in Mexico and 0.7 million individuals in Nuevo Leon); (3) and state, oil, army, and navy workers (14 million individuals in Mexico and 0.3 million individuals in Nuevo Leon) have their own social security institutions and healthcare delivery mechanisms. In contrast, the private healthcare sector consists of individuals who own private insurance (approximately 3% of the total population) and small businesses that provide insurance to their employees (approximately 2% of the total population). Regarding socioeconomic status, approximately 60% of the residents in Monterrey are in the middle class, with an average monthly salary of $1,155 [16].

### Survey design

The survey was designed to evaluate the following: (1) the number of children cared for, healthcare, and type of education; (2) whether the respondents knew a child diagnosed with cancer and their relationship with him/her; (3) knowledge regarding several childhood cancer facts; (4) knowledge about the signs/symptoms of childhood cancer; (5) early diagnostic strategies; and (6) exposure to reliable information about cancer in children and adolescents. The performance of this survey was evaluated in a pilot study involving 30 parents and caregivers in a waiting room of a private children’s outpatient clinic located in Monterrey, which is a reference clinic in the metropolitan area, having a high level of pediatric clinical expertise. Parents and caregivers were chosen and invited while waiting for their child’s consultation. The questions’ adequacy was evaluated using the Delphi technique [17] with a panel of nine experts in Pediatric Oncology.

### Survey tool

The questionnaire included 29 items. Eight questions were designed to obtain the demographic characteristics of the respondents. Four questions were designed to obtain the following information: (1) the number of children cared for, (2) physician responsible for evaluating children, (3) physician’s practice type, and (4) type of school attended by children. Furthermore, two questions regarding the experience of knowing a child diagnosed with cancer and their relationship with him/her were included. Six questions were designed to evaluate knowledge regarding the following aspects: (1) definition of cancer, (2) first thought when hearing the word “cancer,” (3) the most common type of childhood cancer, (4) how frequent they think childhood cancer is, (5) the causes of cancer in children, and (6) the most common pediatric cancer age group. Of these six questions, all but the third and fourth ones were answered using a 5-point Likert scale that measures the level of agreement/disagreement. Three questions about children’s and adolescent’s cancer symptoms were included: (1) warning signs/symptoms (using a 5-point Likert scale measuring the level of agreement/disagreement), (2) whether they consider that any symptom lasting  ≥ 2–3 weeks in children should be suspicious of cancer, and (3) whether they think that Down syndrome is a risk factor for childhood cancer. Two questions were designed to evaluate knowledge about early diagnostic strategies: (1) preventive measures to assure the early diagnosis of childhood cancer (using a 5-point Likert scale measuring the level of agreement/disagreement) and (2) whether they consider that early diagnosis is related to a higher cure rate. The parents’ and caregivers’ exposure to reliable information about pediatric cancer was evaluated by asking the following: (1) whether they had ever received information about childhood cancer, (2) the source of information, (3) knowledge about vaccines that can help prevent certain types of childhood cancer, and (4) whether they wanted to receive information about the warning signs/symptoms, early diagnosis, and treatment of cancer in children.

### Study design and participants

The survey was written in Spanish in Google Forms and distributed to 112 parents and caregivers; each interview was completed in approximately 15 min. Restrictions were made for each question so that responses could not be sent to the final database if there were missing answers. Only complete records were processed; no surveys were excluded. Participation in the study was voluntary, responses were anonymous, and no incentives or compensations were offered. Before the initiation of the study, permission was obtained from the forum concerned. The questionnaire and methodology for this study were approved by the Institutional Human Research and Ethics Review Boards of the Escuela de Medicina y Ciencias de la Salud, Tecnológico de Monterrey (number P000112-LLA2020-CEIC-CR003), and all methods were performed according to relevant guidelines and regulations. Before obtaining their consent, all parents and caregivers were provided with information about the study, concerning its objective, plan, and benefits.

### Statistical analysis

Quantitative variables are reported as medians and interquartile ranges (25^th^ to 75^th^ percentiles) and were analyzed using the Mann–Whitney U test. Categorical variables were analyzed using Fisher’s exact test or the chi-square test. In addition to descriptive analysis, we arranged the survey records from the respondents with experience of knowing a child diagnosed with cancer and compared them with those without this experience. *P* values of ​​less than 0.05 were used to denote statistical significance, and the alpha reliability of all questions was adequate (α = 0.899). The survey was analyzed using Statistical Package for the Social Sciences, version 27.0.

## Results

Of the parents and caregivers in this study, 61.6% were mothers, 29.5% were between the ages of 30 and 34 years, 59.8% had a professional degree, and 77.7% stated that the physician responsible for evaluating their children at least once a year was a pediatrician (Table 1).

**Table 1:** Demographic characteristics of Parents/Caregivers, health care and educational characteristics of children taking care of

		n	%
Total		112	100
Role	Mother	69	61.6
	Father	23	20.5
	Familiar caregiver (grandparent, grandmother, uncle, aunt, cousin)	17	15.2
	Non-familiar caregiver	3	2.7
Gender	Female	88	78.6
	Male	24	21.4
Age range	< 20	5	4.5
	20–24	8	7.1
	25–29	8	7.1
	30–34	33	29.5
	35–39	25	22.3
	40–44	15	13.4
	45–49	9	8.0
	≥ 50	9	8.0
Educational status	None	1	0.9
	Incomplete Primary School	2	1.8
	Complete Primary School	1	0.9
	Incomplete Middle School	0	0
	Complete Middle School	10	8.9
	Incomplete High School	2	1.8
	Complete High School	5	4.5
	Professional Degree	67	59.8
	Master’s Degree	21	18.8
	Doctorate Degree	3	2.7
Marital status	Single	13	11.6
	Married	84	75.0
	Free union	11	9.8
	Separated	3	2.7
	Divorced	1	0.9
	Widowed	0	0
Working status	Employed	80	71.4
	Unemployed	32	28.6
Smoking experience	Smoker	25	22.3
	Non-smoker	87	77.7
Chronic illness status	Diabetes mellitus	9	8.0
	Hypertension	16	14.3
	Cancer	1	0.9
	None	82	73.2
	Other	4	3.6
Number of children cared for	1	31	27.7
	2	43	38.4
	3	31	27.7
	4	1	0.9
	≥ 5	6	5.4
Physician responsible of evaluating children at least once a year	General Physician	16	14.3
	Family Physician	7	6.3
	Pediatrician	87	77.7
	Alternative Medicine practitioner	0	0
	Children taking care of do not get any evaluations with any physician	2	1.8
Physician’s practice type	Public healthcare	16	14.3
	Private healthcare	94	83.9
	None	2	1.8
Type of daycare/school attended by children taking care of	Public daycare/school	15	13.4
	Private daycare/school	78	69.6
	Does not attend daycare/school yet	19	17

**Fig. 1:**
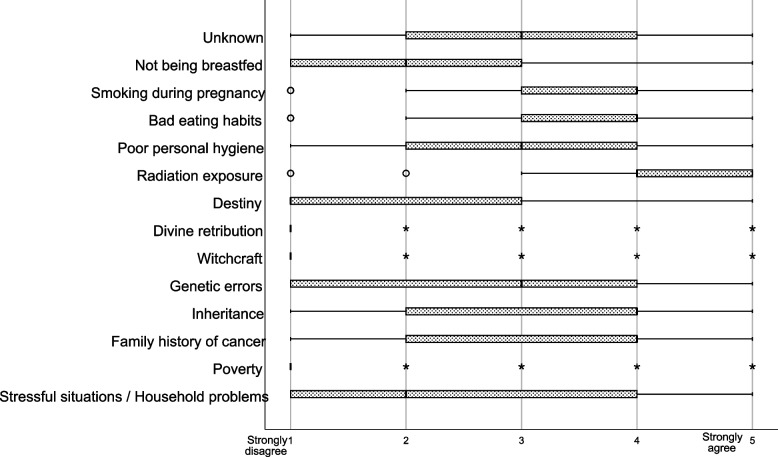
The parents’ and caregivers’ perceptions of the causes of cancer in children. The respondents were instructed to rank several causes of cancer in children. The line in the middle of the box represents the median; the edges of the box represent the 25^th^ and 75^th^ percentile interquartile ranges; the whiskers represent the minimum and maximum observations

Among the 112 parents and caregivers, 41.1% knew a child diagnosed with cancer. Regarding the definition of cancer, 83.9% strongly agreed that it is a worrisome disease, and 71.4% strongly agreed that it is a painful illness. The question regarding the first thought parents and caregivers had when hearing the word “cancer” showed that 64.3% strongly agreed is “suffering,” and 62.5% strongly agreed is “chemotherapy.” More than 90% mentioned leukemia as the most common type of cancer in children, and 50.9% stated that childhood cancer is frequent. Moreover, 20.5% strongly agreed the most common pediatric cancer age group is the 6–12 years year group (Table 2). Among the provided options for the causes of cancer in children, the most highly ranked option was radiation exposure (Fig. 1).

**Table 2:** Respondents’ history of knowing a child diagnosed with cancer, the relationship with him/her, and knowledge regarding childhood cancer facts

	n	%
Total	112	100
Experience of knowing a child diagnosed with cancer		
Yes	46	41.1
No	66	58.9
Relationship with the child with cancer		
Relative	10	8.9
Friend/neighbor	21	18.8
I have seen children with cancer in the hospital/on the street/on TV	9	8.0
I have seen children with cancer on social networking sites	7	6.3
I have never met a child with cancer	65	58
Definition of cancer		
A fatal disease		
Strongly disagree	9	8.0
Disagree	13	11.6
Neutral	14	12.5
Agree	29	25.9
Strongly agree	47	42.0
A worrisome disease		
Strongly disagree	6	5.4
Disagree	0	0
Neutral	3	2.7
Agree	9	8.0
Strongly agree	94	83.9
A painful illness		
Strongly disagree	5	4.5
Disagree	3	2.7
Neutral	3	2.7
Agree	21	18.8
Strongly agree	80	71.4
A disease that always causes death		
Strongly disagree	34	30.4
Disagree	25	22.3
Neutral	16	14.3
Agree	29	25.9
Strongly agree	8	7.1
I do not know what cancer is		
Strongly disagree	74	66.1
Disagree	18	16.1
Neutral	4	3.6
Agree	10	8.9
Strongly agree	6	5.4
First thought when hearing the word “cancer”		
Very bad pain		
Strongly disagree	5	4.5
Disagree	12	10.7
Neutral	17	15.2
Agree	27	24.1
Strongly agree	51	45.5
Suffering		
Strongly disagree	5	4.5
Disagree	5	4.5
Neutral	4	3.6
Agree	26	23.2
Strongly agree	72	64.3
Hair loss		
Strongly disagree	6	5.4
Disagree	2	1.8
Neutral	14	12.5
Agree	38	33.9
Strongly agree	52	46.4
Chemotherapy		
Strongly disagree	5	4.5
Disagree	1	0.9
Neutral	8	7.1
Agree	28	25.0
Strongly agree	70	62.5
Face with mask		
Strongly disagree	16	14.3
Disagree	23	20.5
Neutral	35	31.3
Agree	18	16.1
Strongly agree	20	17.9
Death		
Strongly disagree	5	4.5
Disagree	22	19.6
Neutral	24	21.4 28.6
Agree	32	
Strongly agree	29	25.9
Being all time in the hospital		
Strongly disagree	11	9.8
Disagree	22	19.6
Neutral	33	29.5
Agree	27	24.1
Strongly agree	19	17.0
Most common type of cancer in children		
Leukemia	104	92.9
Lymphoma	5	4.5
Brain tumors	3	2.7
Frequency of childhood cancer		
Very frequent	25	22.3
Frequent	57	50.9
Occasional	0	0
Rare	30	26.8
Cancer does not exist in children	0	0
Most common pediatric cancer age group		
0-1 months		
Strongly disagree	30	26.8
Disagree	18	16.1
Neutral	48	42.9
Agree	9	8.0
Strongly agree	7	6.3
2 months – 2 years		
Strongly disagree	16	14.3
Disagree	15	13.4
Neutral	44	39.3
Agree	24	21.4
Strongly agree	13	11.6
3–5 years		
Strongly disagree	7	6.3
Disagree	4	3.6
Neutral	43	38.4
Agree	40	35.7
Strongly agree	18	16.1
6–12 years		
Strongly disagree	6	5.4
Disagree	6	5.4
Neutral	38	33.9
Agree	39	34.8
Strongly agree	23	20.5
13–18 years		
Strongly disagree	13	11.6
Disagree	10	8.9
Neutral	41	36.6
Agree	29	25.9
Strongly agree	19	17.0

**Fig. 2:**
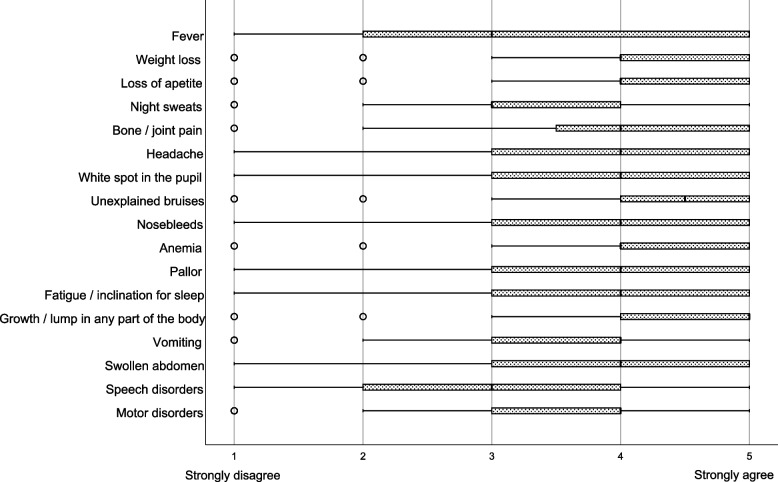
Distribution of the parents’ and caregivers’ statements about the warning signs and symptoms of cancer in children. The respondents were instructed to rank several signs and symptoms of cancer in children. The line in the middle of the box represents the median; the edges of the box represent the 25^th^ and 75^th^ percentile interquartile ranges; the whiskers represent the minimum and maximum observations

A question was asked to determine which signs/symptoms the parents and caregivers considered a warning sign or symptom of childhood cancer (Fig. 2). The three most highly ranked options were growth/lump in any part of the body, unexplained bruises, and weight loss. Among the respondents, 58.9% answered that any symptom lasting  ≥ 2–3 weeks in children should be suspicious for cancer, and 88.4% did not consider Down syndrome as a risk factor for cancer.

**Fig. 3:**
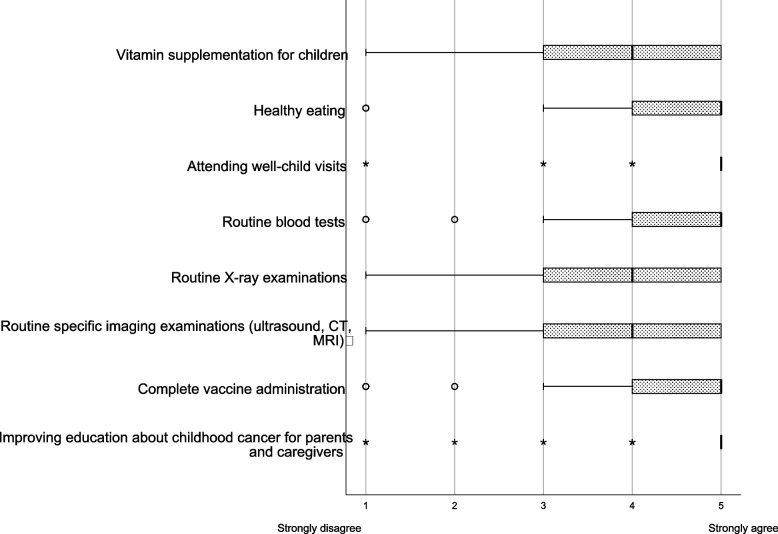
Perceptions of the parents and caregivers on the preventive measures to ensure the early diagnosis of childhood cancer. The respondents were instructed to rank several preventive measures for the early diagnosis of cancer in children. The line in the middle of the box represents the median; the edges of the box represent the 25^th^ and 75^th^ percentile interquartile ranges; the whiskers represent the minimum and maximum observations

Almost all respondents (97.3%) considered that the early diagnosis of cancer in children is related to a higher cure rate (Table 3), and the three most highly ranked options regarding the preventive measures they considered important to ensure the early diagnosis of childhood cancer were as follows: improving education about childhood cancer for parents and caregivers, attending well-child visits, and healthy eating (Fig. 3).

**Table 3:** Parents’/caregivers’ statements about childhood cancer symptoms, early diagnosis strategies, and exposure to information about cancer in children

	n	%
Total	112	100
Opinion about any symptom lasting more than 2 to 3 weeks (including fever) in children should be suspicious for cancer		
Yes	66	58.9
No	46	41.1
Down syndrome is a risk factor for childhood cancer		
Yes	13	11.6
No	99	88.4
An early diagnosis of cancer in children is related to a higher cure rate		
Yes	109	97.3
No	3	2.7
Exposure to information about childhood cancer		
Yes	38	33.9
No	74	66.1
Source of information about cancer in children		
Television		
Yes	25	22.3
No	87	77.7
Movies		
Yes	9	8.0
No	103	92.0
My children’s school		
Yes	7	6.3
No	105	93.8
My own school		
Yes	6	5.4
No	106	94.6
Pediatrician		
Yes	26	23.2
No	86	76.8
Other healthcare professional (nurse, general physician, family physician)		
Yes	22	19.6
No	90	80.4
Relative/Neighbor		
Yes	11	9.8
No	101	90.2
Newspaper/Magazines		
Yes	13	11.6
No	99	88.4
Internet		
Yes	32	28.6
No	80	71.4
Social networking sites		
Yes	23	20.5
No	89	79.5
Knowledge about vaccines that can help prevent certain types of childhood cancer		
Yes	29	25.9
No	83	74.1
Willingness to receive reliable information about warning signs and symptoms, early diagnosis and treatment of cancer in children		
Yes	104	92.9
No	8	7.1

An analysis of the parents’ and caregivers’ exposure to information about childhood cancer showed that 66.1% had never received information. More than 70% stated that they did not know that there are vaccines that can help prevent certain types of childhood cancer; and 92.9% expressed the desire to receive information about the signs/symptoms, early diagnosis, and treatment of cancer in children (Table 3).

The following relationships were found to be statistically significant, considering the two groups regarding knowing/not knowing a child with cancer (data not shown): defining cancer as a fatal disease (W = 1073.50, *p* = 0.006); defining cancer as a disease that always causes death (W = 1060.00, *p* = 0.005), with the group that do not know a child with cancer more likely to refer to both definitions; vitamin supplementation for children (W = 1053.00, *p* = 0.005) and getting routine specific imaging examinations done (i.e., ultrasonography, computed tomography, and magnetic resonance imaging) (W = 1052.50, *p* = 0.004) as preventive measures to assure the early diagnosis of childhood cancer; exposure to any information about childhood cancer (X^2^ = 8.994; df = 1; *p* = 0.003), pediatricians as the source of information about cancer in children (X^2^ = 3.865; df = 1; *p* = 0.049), and knowledge about vaccines that can help prevent certain types of childhood cancer (X^2^ = 7.129; df = 1; *p* = 0.008).

## Discussion

Continued medical advancements have demonstrated that childhood cancer cannot be prevented. Therefore, the cornerstone for improving outcomes using more effective and less toxic treatments relies on several factors, of which early diagnosis is of great importance. However, the pathway toward better outcomes for children with cancer among LMICs is often impeded by numerous obstacles, with a timely diagnosis relying on multiple factors: cancer biology, patient characteristics, physician experience/education, access to healthcare services, and children’s family environment [4, 18]. However, there is a need to focus on modifiable factors, such as knowledge of childhood cancer in parents and caregivers, to help diagnose this group of diseases earlier. For this reason, this study was designed to evaluate the parents’ and caregivers’ knowledge about this topic to have a better understanding of the influence of this factor in the delay of pediatric cancer diagnosis; thus we can focus on an intervention to implement effective programs and public policies to ensure the timely diagnosis of cancer in children.

To the best of our knowledge, few studies in Latin America have assessed what parents and caregivers know about childhood cancer [4, 7], and two studies of this kind were found, which were conducted in Turkey [19] and Greece, respectivey [20]. Evaluating parents’ and caregivers’ current knowledge of cancer is important because parent-related factors, such as lower educational level, younger age, and lower socioeconomic status, can be associated with a greater diagnostic delay of cancer in pediatric patients [4, 5]. In this study, more than half of the respondents had a professional degree, and 29.5% were between the ages of 30 and 34 years. However, 66.1% stated that they had never received any information about childhood cancer, indicating that even though more than half of them had a high educational level, there is a need for better education regarding pediatric cancer.

More than 75% of the parents and caregivers stated that the physician responsible for evaluating their children at least once a year was a pediatrician, indicating the importance of regular well-child visits to establish continuous care and surveillance of all children, which can help identify subtle symptoms that may not be noted initially as important by the children’s families [21].

Several parents and caregivers reported that they knew a child with cancer, whereas the top definitions of cancer that the respondents strongly agreed to were that it is a worrisome and painful disease; and when the respondents heard the word “cancer,” most of them thought of “suffering.” These findings highlight how parents and caregivers perceive this disease, and although cultural background must always be considered [22], cancer in children is mostly identified as the most frightening illness. An analysis of contemporary representations of childhood cancer in Romanian media [23] reported that a diagnosis that can lead to premature death, such as cancer, is perceived as a transgression that often inspires negative reactions, sentiments, and stigma. This could support this study’s findings where the respondents perceived that childhood cancer is worrisome and painful and the overall association of cancer with words, such as “suffering.”

The incidence of childhood cancer varies among different countries; particularly, in Mexico, it increased from 133.5/million children in 2007 to 150.1/million children in 2015 [24]. Moreover, although the most prevalent cancer may also vary, in Mexico, acute lymphoblastic leukemia (ALL) takes the lead [24]. In this study, the most common type of childhood cancer that the respondents reported was leukemia; 20.5% of the respondents strongly agreed that the most common age group affected by cancer is the 6–12 years year group. These findings suggest that although they are aware of the most common type of childhood cancer, the respondents lack knowledge regarding the most common age group they must keep a close eye on because the age at which the incidence of pediatric cancer is the highest is between 2 and 6 years [24].

When the respondents were asked to determine which options they considered as the causes of childhood cancer; the top three responses with the highest ranks of respondents who strongly agreed were radiation exposure (52.0%), smoking during pregnancy (27.0%), and family history of cancer along with bad eating habits (both with 20.0%). Since ALL is the most prevalent type of childhood cancer in Mexico, it is imperative that parents and caregivers know which factors can be related to its etiology. Several factors might contribute to the etiology of ALL or might have a protective effect against this disease, including paternal smoking [25, 26] and breastfeeding [27, 28], respectively. The parents and caregivers in this study showed a lack of knowledge regarding the protective effect breastfeeding might have against childhood cancer, particularly ALL, as only a few strongly agreed that lack of breastfeeding might be a cause of cancer in children, in almost an even proportion to witchcraft and divine retribution.

A Mexican multicenter cohort study showed that the average time from the onset of symptoms to the diagnosis of childhood cancer was 43.5 ± 22.5 days [29]. Heightened recognition of the early signs/symptoms among the general population and primary care providers is essentially the first factor in a chain of events that ultimately lead to a child’s prompt diagnosis. In this study, the top three signs and symptoms that the respondents strongly agreed to be considered a warning sign/symptom of childhood cancer were growth/lump in any part of the body, unexplained bruises, and weight loss. The literature highlights that the most common symptoms of cancer in children are as follows: (1) pallor, fatigue, and malaise; (2) fever; and (3) recurrent or treatment-resistant infections [30]. Although all signs/symptoms included in this question should be considered a warning and even though results showed that respondents have some idea of which symptoms are more common, there is still a low level of knowledge considering that they did not even mention fever as one of the top three signs/symptoms of childhood cancer, and that the biggest challenge in diagnosing this group of diseases is that they often present with non-specific symptoms that less frequently have serious outcomes.

Among the respondents, 58.9% answered that any symptom lasting  ≥ 2–3 weeks should be suspicious of cancer. This is remarkable as it has been reported that the average time from symptom onset and the first medical appointment received was 9.2 ± 16.6 days, followed by 34.3 ± 16.3 days from the initial appointment to the diagnostic confirmation of cancer, after an average of 2.3 medical consults [29], highlighting the need for searching medical care as soon as parents and caregivers detect persistent symptoms. A qualitative study of parents with children newly diagnosed with cancer showed that most of them initially attributed their child’s symptoms to minor illnesses, particularly when the latter was coupled with normal behavior, interpreted as “incompatible” with cancer [31].

An important contributing factor to mortality in children with cancer in Mexico is diagnosis at advanced disease stages, estimated to happen in almost 70% of cases [11], in contrast to high-income countries, where approximately 70% of cases are diagnosed with local or regional disease [32]. Among the respondents, 97.3% considered that the early diagnosis of cancer in children is related to a higher cure rate and that the three top preventive measures were as follows: improving education about childhood cancer in parents and caregivers, attending well-child visits, and healthy eating. Furthermore, 92.9% of the respondents expressed the desire to receive information about childhood cancer. Although most respondents were aware of the important role of the early diagnosis of pediatric cancer in achieving better outcomes, the results also highlighted that the respondents have the desire to receive trustable information, emphasizing the deficiency of knowledge of childhood cancer.

This study has limitations, including a limited number of respondents. Moreover, those willing to respond may not be representative of the entire population of Mexican parents and caregivers. It could also be possible that the characteristics of the parents and caregivers who responded may be different from those of non-respondents, which might have affected the study results and interpretations. Besides, this study does not address the perceptions of parents and caregivers on the communication about childhood cancer from different sources of information or other cultural factors that can influence the knowledge they have about this disease.

Regarding the next steps following this study, it would be interesting to replicate the distribution of the survey and increase the sample size to have a more balanced and representative group of respondents of the entire population of Mexican parents and caregivers, including those of different geographic areas in Mexico and educational level and those receiving not only private but also public healthcare. This would help generalize the results to the general Mexican population.

## Conclusions

Although a long pathway of factors is involved toward achieving the timely and optimal diagnosis of cancer in children, strengthening the use of preventive childhood healthcare services and public campaigns to enhance parents’ and caregivers’ knowledge of childhood cancer is mandatory to decrease the delay interval between symptom presentation and the diagnosis of cancer in children, to achieve better outcomes with fewer complications. In the future, the use of tools such as applications that help educate more parents and caregivers, will strengthen the knowledge and contribution regarding this issue because educating the population is of vital importance for the prompt diagnosis of the disease. Finally, the development of public policies that support the early diagnosis of cancer, particularly in children, who is a doubly vulnerable population, must be promoted.

## Data Availability

The datasets used and analyzed during the current study are available from the corresponding author (J.R.D.I.) on reasonable request.
